# Antibacterial and Angiogenic Poly(ionic liquid) Hydrogels

**DOI:** 10.3390/gels8080476

**Published:** 2022-07-28

**Authors:** Chengju Sheng, Xuemei Tan, Qing Huang, Kewen Li, Chao Zhou, Mingming Guo

**Affiliations:** 1 School of Chemistry and Chemical Engineering, Southwest University, Chongqing 400715, China; scj1995@email.swu.edu.cn (C.S.); xm066248@163.com (X.T.); qinghuang3602@163.com (Q.H.); 2 College of Materials and Environmental Engineering, Hunan University of Humanities, Science and Technology, Loudi 417000, China; likewen4310@163.com; 3 Institute of Biomedical Engineering and Health Sciences, Changzhou University, Changzhou 213164, China

**Keywords:** antibacterial hydrogels, imidazolium poly(ionic liquids), vascular repair, anti-inflammatory, deferoxamine

## Abstract

Wounds, particularly under low-hydration conditions, require more time to repair successfully. Therefore, there is an urgent need to develop wound dressings that can accelerate wound healing. Hydrogels, which can maintain a moist environment around the wound and allow gas to pass through the material, act as antibacterial hydrogels as dressings and have great application value in the treatment of wounds. In addition, wound dressings (hydrogels) containing antibacterial capacity have lasting antibacterial effects and reduce damage to cells. In this work, we firstly synthesized two antibacterial agents: imidazolium poly(ionic liquids) containing sulfhydryl (Imidazole-SH) and ε-Poly(lysine) containing SH (EPL-SH). Then, lysine as a cross-linking agent, by “thiol-ene” click reaction, was mixed with Deferoxamine (DFO) to prepare the antibacterial hydrogels. The in vitro assays showed that the hydrogels could effectively kill *Escherichia coli* (*E. coli*) and *Staphylococcus aureus* (*S. aureus*). In addition, it also could reduce the inflammatory response produced by Lipopolysaccharide (LPS). More importantly, according to the transwell and angiogenesis assays, DFO-incorporated hydrogels promoted the migration and vascular repair of human umbilical vein endothelial cells (HUVECs). All the results revealed that the hydrogels provided new strategies for wound dressings.

## 1. Introduction

Wound healing faces many challenges in clinical medicine, such as harsh wound environment, prolonged inflammation, reduced angiogenesis, abnormal re-epithelialization, etc. [1,2]. It is necessary to build a physical barrier to protect wounds against water loss and infection [3,4]. Therefore, hydrogel, a three-dimensional hydrophilic network material, is a good choice because of its strong water absorption, water retention, and mechanical strength [5]. Traditional hydrogel wound dressings (e.g., gauzes [6], absorbent cotton [7], and bandages [8]) have been widely used in clinical treatments, but their poor biological properties limit their application [9]. In addition, some researchers provided a chitosan–Vaseline gauze and a nano-silver sodium alginate dressing to accelerate the healing of wounds [10,11]. However, these substances did not provide a wet environment to promote wound healing due to their poor water retention [12,13]. Alginate (SA) and Chitosan (CS) could form hydrogels by the cross-linking of metal ions (Ca^2+^, Cu^2+^ and Ag^2+^ et al.) and showed good antibacterial ability against both *E. coli* and *Pseudomonas* *aeruginosa* bacteria [14,15]. However, the loaded Ag NPs were unstable, and the released Ag ions showed obvious toxicity to normal cells and tissues [16]. Photothermal (PTT) antibacterial hydrogels were an effective strategy to treat bacterial infections using hyperthermia generated by photothermal materials under visible or near-infrared light irradiation [15,17]. These hydrogels could convert light into heat mainly by relying on noble metal materials [18], 2D nanomaterials such as black phosphorus (BP) nanosheets [19], and transition metal sulfides (molybdenum disulfide (MoS_2_)) [20] et al.

Since the first synthesis of quaternary ammonium salts by Jocobs et al. in 1915, its antibacterial properties have been gradually discovered, which is also the first proposal of generalized poly (ionic liquids) (PILs) antibacterial agents [21,22]. The antibacterial mechanism is the positively charged ionic liquids (ILs) that attract the negatively charged bacterial cell walls through the anion–cation interactions [23,24]. Imidazolium PILs are one of the most widely used ILs, which have potent broad-spectrum bactericidal activity and slight cell toxicity [25]. ε-poly (lysine) (EPL) is a natural polypeptide antibacterial agent [26]. It not only has a significant inhibitory effect on *S. aureus* and *E. coli* but also has high water solubility, high thermal stability, and low toxicity [27,28].

During the process of wound healing, vascular repair is an extremely important step. Hemostatic activation, including vasoconstriction and clot formation occurs after tissue damage during normal wound healing [29]. Blood platelets secrete growth factors and proinflammatory generating fibrin clots in the wound area [30,31]. Fortunately, deferoxamine (DFO) is an iron chelator that the FDA (Food and Drug Administration) approved for clinical use, which could advance the formation of new blood vessels under normal and pathological conditions and promote the expression of HIF-1α and its downstream gene-VEGF [32,33]. 

**Figure 1:**
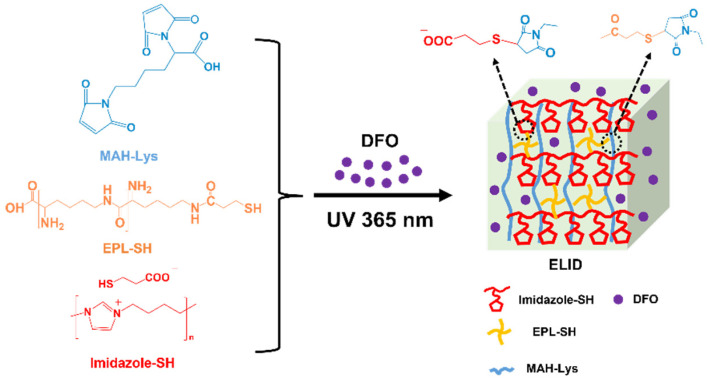
Schematic presentation for the synthesis of ELID hydrogels.

In the present study, we developed a therapeutic strategy based on imidazolium PILs hydrogel with DFO for improving angiogenesis to accelerate wound healing. Firstly, the antibacterial imidazolium PILs containing sulfhydryl (SH) (Imidazole-SH) and ε-Poly(lysine) containing SH (EPL-SH) were synthesized as per previous methods. In addition, they were cross-linked with lysine-grafted maleic anhydride (MAH-Lys) via a “thiol-ene” click reaction (Figure 1) [34]. The above-mentioned “click” chemical reactions have proven to be good candidates for cross-linking reactions for the preparation of hydrogels because of their high efficiency and selectivity, which can precisely engineer and enable the formation of hydrogels with tunable properties [35]. Subsequently, the physical, antibacterial, hemolytic (the key index of hemocompatible), and anti-inflammatory activities of hydrogels were evaluated. In particular, a reduction in inflammatory cells results in lower levels of cytokines, which in turn reduce the production of tissue-degrading proteases and lead to a reduction in the likelihood of long-term inflammation, which aids in the healing process. The results demonstrated that hydrogels prepared by this strategy could provide a new avenue for wound healing.

## 2. Results and Discussion

### 2.1. Properties of Hydrogels

The hydrogels were fabricated with Imidazole-SH, EPL-SH and MAH-Lysr via a “thiol-ene” click reaction, which is named ELI. The hydrogels prepared from DFO were named ELID. As controls, hydrogels without Imidazole-SH and DFO were named EL.

**Figure 2:**
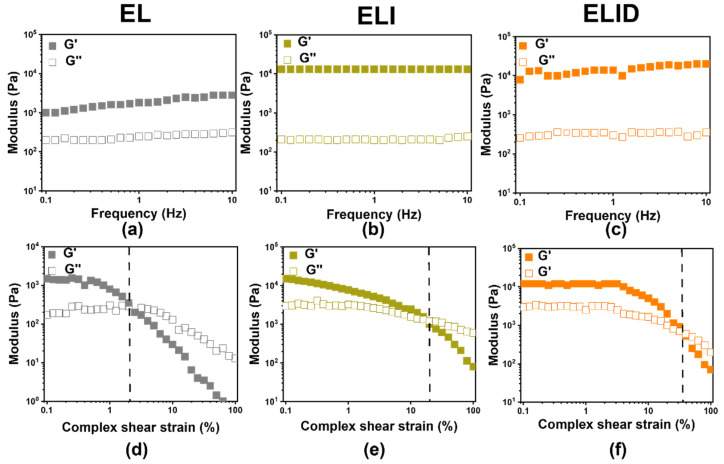
The frequency sweep (**a**–**c**) at *γ* = 0.5% constant oscillatory strain and *f* = 0.1–10 Hz; amplitude sweep (**d**–**f**) oscillatory strain (0.1–100%) at a constant frequency (*f* = 1 Hz) (the dotted lines are fractured point) (*n* = 3).

The mechanical property of the hydrogels was one of the significant factors for wound dressing, which could be assessed by oscillatory rheometry [36]. The frequency sweep results shown in Figure 2a–c demonstrated that the G′ (storage modulus) modulus of all the hydrogels was higher than G″ (loss modulus), especially ELI and ELID having the highest G′ (1.4 × 10^4^ Pa) and G″ (3.5 × 10^2^ Pa), which was not low compared to the modulus (G′ and G″) of other hydrogels [37]. In addition, the amplitude sweep indicated that ELI and ELID were fractured at approximately 20% and 35%, respectively, which is higher than EL (2%), suggesting the EL was brittle. This was because Imidazole-SH and EPL-SH chemically cross-linked with MAH-Lys to form semi-inter penetrate network (semi-IPN) hydrogel and provided more SH groups to promote “thiol-ene” click reaction, thereby increasing the cross-link density of hydrogels [35,38].

The freeze-dried hydrogels were used with uniaxial compression measurements to evaluate their mechanical properties (Figure S4a). The results showed that the ELI (22.1%) and ELID (21.2%) had higher rupture compression stress than EL (15.7%). It could also demonstrate that Imidazole-SH penetrated the hydrogels and increased its mechanical properties [39].

### 2.2. Antibacterial and Antibiofilm Activities of Hydrogels

**Figure 3:**
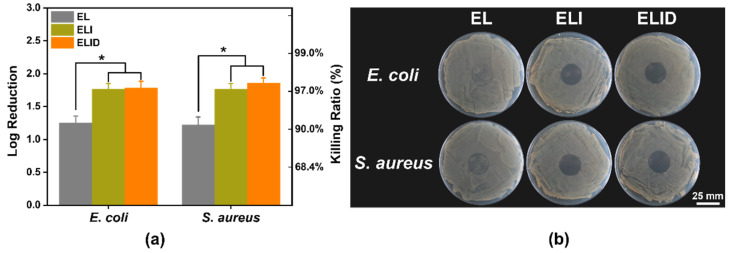
(**a**) In vitro antibacterial efficiency of EL, ELI, and ELID (PBS as blank); (**b**) contact-active antimicrobial activity (*E. coli* and *S. aureus*) of hydrogels (*n* = 4, * means *p* ≤ 0.05).

The *S. aureus* (10^7^ CFU/mL) and *E. coli* (10^6^ CFU/mL) were treated with hydrogel dressings (EL, ELI, and ELID). The antibacterial properties of hydrogels after treatment for 4 h are shown in Figure 3a, displaying that ELI and ELID could effectively kill bacteria, including *E. coli* (95.4% and 96.2%) and *S. aureus* (97.2% and 97.4%) compared with EI (33.5% and 30.8%). It can be found that the addition of Imidazole-SH increased the antibacterial efficiency of the hydrogel dressing. Due to the Gram-positive bacteria’s thick cell walls and thick and dense peptidoglycan layer, it is difficult for antibacterial materials to kill it. However, compared to other antibacterial hydrogel dressings, this hydrogel (ELI and ELID) also has better antibacterial properties for Gram-positive *E. coli* [39,40]. Figure 3b shows the contact-active antimicrobial activity of hydrogels.

The results suggested that hydrogels containing PILs prevented growth with very few or no bacterial colonies under the gels, and this activity also prevented the formation of drug-resistant biofilms. Thus, the hydrogels with PILs exhibited strong antibacterial activity.

**Figure 4:**
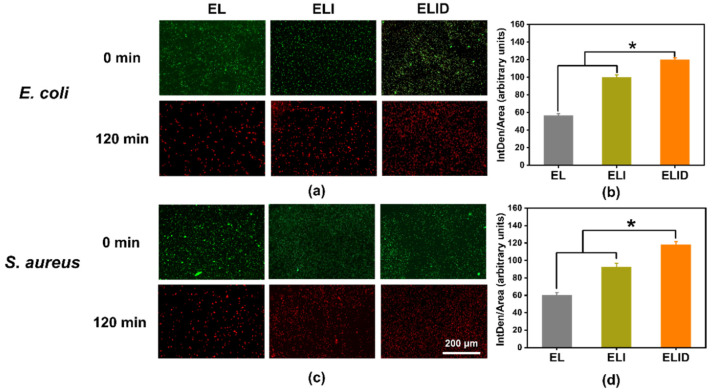
(**a**,**c**) Fluorescence images of LIVE/DEAD-stain biofilms after EL, ELI, and ELID treatment for 120 min; (**b**,**d**) analysis of red fluorescence intensity after EL, ELI, and ELID treatment for 120 min (*n* = 3, * means *p* ≤ 0.05).

The antibiofilm activities of hydrogels were evaluated by fluorescence images. As shown in Figure 4a,c, the *E. coli* and *S. aureus* biofilms could be contacted by all the hydrogels for 2 h, as indicated by a decrease of green-fluorescent-labeled bacteria signals, which was consistent with the lower CFU count of the antibiofilm assay. Interestingly, the red fluorescence of the hydrogels (ELI and ELID) significantly changed before and after light exposure. This phenomenon was due to the addition of imidazole-SH improving the antibacterial ability of hydrogels, and DFO alone was not sufficient to kill the bacteria [34]. Figure 4b,d shows an analysis of the red fluorescence intensity (measured by ImageJ v1.8.0 software), which indicated that ELI and ELID had obvious antibacterial advantages over *E. coli* and *S. aureus* in comparison to EL.

**Figure 5:**
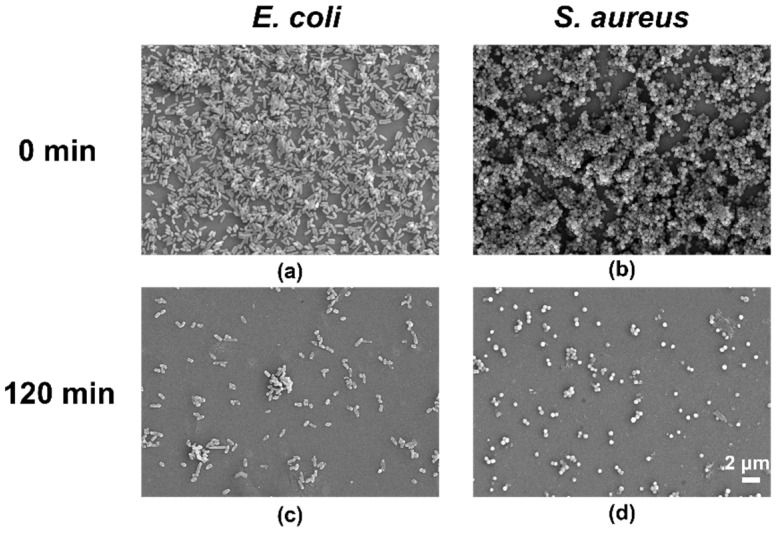
FE-SEM images of *E. coli* (**a**) and *S. aureus* (**b**) cultivated for 24 h. (**c**,**d**) Images after treatment with hydrogel ELID (*n* = 3).

The FE-SEM showed similar results to the fluorescence images. After treatment with ELID for 2 h, the *E. coli* and *S. aureus* biofilms disappeared, suggesting that most of the bacteria were dead (Figure 5). The above results revealed that the hydrogels containing Imidazole-SH could penetrate quickly into *E. coli* and *S. aureus* biofilm within 2 h, which is likely due to the strong electrostatic interactions [41].

### 2.3. In Vitro DFO Release and Hemolysis Assay of Hydrogels

DFO, an FDA-approved medicine, could chelate redundant Fe^2+^ in blood and spleen and could accelerate vessel formation in wound healing and bone regeneration [42]. In recent years, its dramatic angiogenesis function has attracted increasing attention. Chen et al. designed a novel drug delivery system by DFO, but there were few reports on DFO-loaded hydrogels with antibacterial functions that could synergize antibacterial and vascular repair [32,42]. In this work, we designed DFO-loaded ELID hydrogels, which released 80% of the DFO content in 24 h (Figure S4c). This release rate, combined with previous antibacterial function, facilitated wound healing. This was because these hydrogels could effectively act as an antibacterial agent first and then revascularize, thereby promoting tissue wound repair. 

Hemolysis is a key indicator for evaluating the biocompatibility of hydrogels [43]. As shown in Figure S4b, all of the hemolysis rates of the hydrogels at 12 h were 0.23 ± 0.03%, which is less than the international permeation level of 5% for biomaterials [44].

### 2.4. In Vitro Transwell and Angiogenesis Assay of Hydrogels

**Figure 6:**
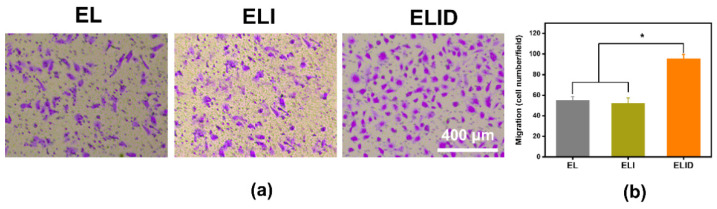
(**a**) HUVECs transwell assay of EL, ELI, and ELID (the scale bar = 400 μm); (**b**) HUVECs migration number of fields for EL, ELI, and ELID (PBS as blank and *n* = 4, * indicates *p* ≤ 0.05).

The scratch test is a commonly used method to study cell migration in vitro, which simulates the process of cell migration in vivo to a certain extent, while the transwell test can detect both cell migration ability and invasion ability [45]. This experimental result proves more than the scratch result that this hydrogel (ELID) has better application potential [39]. In this work, a transwell assay was performed to assess the impact of DFO on HUVEC migration [45]. The results showed that the DFO derived from the hydrogels (ELID) significantly enhanced the migratory ability of HUVECs (Figure 6a). The cell migration number of fields for ELID (95) was higher than that of EL (55) and ELI (Figure 6b) (54). 

**Figure 7:**
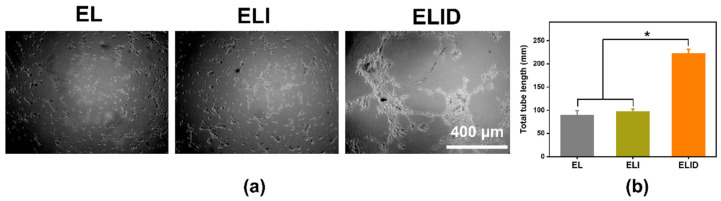
(**a**) The tube formation assay of EL, ELI, and ELID (the scale bar = 400 μm); (**b**) the total tube length of EL, ELI, and ELID (PBS as blank and *n* = 4, * means *p* ≤ 0.05).

Angiogenesis benefits the removal of debris and prepares the wound bed, and the endothelial cells interacted with the associated extracellular matrix and regulate the wound-related angiogenesis [33]. We used HUVECs as the model cells to measure the HUVECs for angiogenesis. The three-dimensional cell culture, a common assessment tool, could enable three-dimensional growth, which was applied widely in most angiogenesis assessments [33,46,47]. As shown in Figure 7, the total tube length of ELID (223 nm) was longer than EL (90 nm) and ELI (97 nm). From the data, it was clear that the neovascularization persisted after 8 h in the DFO-loaded group (ELID) in contrast to that of the free DFO-loaded groups (EL and ELI), and this could be attributed to the sustained and controlled DFO release from the hydrogels (ELID) [48].

### 2.5. In Vitro Anti-Inflammation Activity of Hydrogels

**Figure 8:**
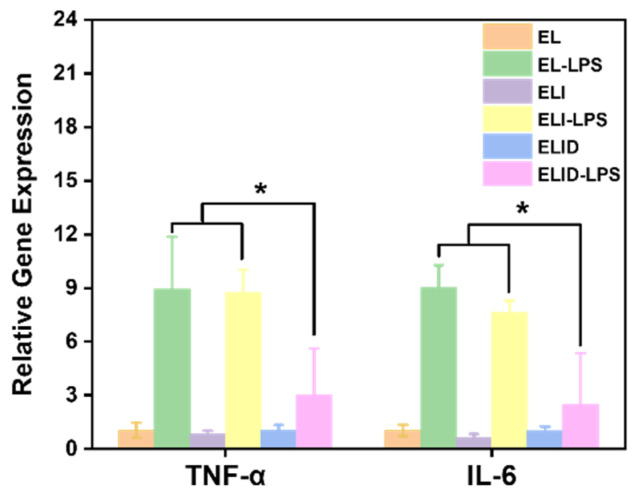
TNF-α and IL6 expression in U-937 cells after being treated with LPS (*n* = 4, * means *p* ≤ 0.05).

DFO can enhance the secretion of anti-inflammatory substances in the body. Park et al. indicated that the tissue pretreated with DFO more efficiently guides and reprograms macrophage polarization into the M2 phase, an anti-inflammatory state [49]. The ELID hydrogel we prepared retained the original DFO vascular and anti-inflammatory function. In the LPS-induced U-937 cells inflammation model, the pro-inflammatory cytokines TNF-α and IL-6 in the control group increased significantly. However, when the cells were pretreated with hydrogels containing DFO, the expression levels of TNF-α and IL-6 (Figure 8) significantly decreased. These results could also prove that DFO as the main component could inhibit the inflammatory cytokine (IL-6 and TNF-α) release in the inflammation cell model [44].

## 3. Conclusions

In summary, EPL-SH and Imidazole-SH were synthesized, and MAH-Lys, as a cross-linker, was a highly effective antibacterial agent. Vascular repair hydrogels were prepared by UV light initiation via the “thiol-ene” click reaction. The hydrogels containing Imidazole-SH (ELI and ELID) had higher mechanical strengths and antibacterial properties compared to EL due to the form of semi-IPN. Additionally, an in vitro assay showed that EPLI could kill *S. aureus* by up to 97.4% and that all the hydrogels had low hemolytic activities. More importantly, the addition of DFO hydrogels had significant anti-inflammatory activities that inhibited the release of various inflammatory mediators by regulating cell signaling pathways and avoiding an inflammatory cascade reaction as well. We believe that these findings provide a novel vision of the design of antibacterial hydrogel dressings, which have a wide range of applications in the biomedical field.

## 4. Materials and Methods

### 4.1. Materials

1,4-diaminobutane (98%), hydrobromic acid (48 wt% in H_2_O), ε-poly (lysine) (EPL, *M*_W_ > 5000 g mol^−^^1^), 1-(3-Dimethylaminopropyl)-3-ethylcarbodiimide methiodide (EDC), N-Hydroxy succinimide (NHS), 3-Mercaptopropionic acid, Maleic anhydride (99%), L-Lysine (98%), 3,3’-Dithiodipropionic acid (99%) and 1,4-Dithiothreitol (DTT, 97%) were purchased from Aladdin, Shanghai, China. Dulbecco’s Phosphate Buffered Saline (PBS), glyoxal (40%), formaldehyde (40%), Sodium ethylate (CH_3_CH_2_ONa), Deferoxaminemesylate salt (DFO, ≥92.5%), and lipopolysaccharide (LPS) were purchased from Sigma-Aldrich, St. Louis Missouri, USA. Endothelial culture medium (ECM), fetal bovine serum (FBS), and the L13152 LIVE/DEAD^®^ Bac Light TM Bacterial Viability Kit were purchased from Thermo Fisher Scientific, Waltham, MA, USA. *E. coli* (DH 5α), *S. aureus* (ATCC 25923), HUVECs, lysogeny broth (LB), Mueller–Hinton broth (MHB), Mueller“°”Hinton agar (MHA), and CCK-8 were purchased from BeyoClick™, Shanghai, China. The 4% paraformaldehyde was purchased from Boster Biological Technology Co., Wuhan, China. 

### 4.2. Synthesis of EPL-SH

The synthesis of EPL-SH was performed following a published method [34]. EPL (1 g, 7.87 mmol), EDC (1.51 g, 7.87 mmol), and NHS (0.91 g, 7.87 mmol) were dissolved in MES pH 5.5 solution (100 mL) at room temperature, and then 3,3′-dithiodipropionic acid (0.83 g, 3.94 mmol) was added, and the reaction solution was stirred for 48 h. The product was obtained after being dialyzed (1 kDa MWCO) and freeze-dried. The above polymer product (2.5 g, 5 mmol) and DTT (0.77 g, 5 mmol) were dissolved in 20 mL of THF and heated to 50 °C in a N_2_ atmosphere for 24 h. The final product was obtained after being dialyzed (1 kDa MWCO) and freeze-dried. ^1^H NMR (500 MHz, D_2_O, d, δ (ppm): 3.86 (s, C=O-C*H*-NH_2_), 3.25 (s, C*H_2_*-NH-C=O-C*H*-NH_2_), 2.75 (s, NH-C=O-C*H_2_*), 1.75 (s, C*H_2_*-SH), 1.5 (s, NH_2_-CH-C*H_2_*-C*H_2_*), 1.26 (s, NH_2_-CH-CH_2_-CH_2_*-CH_2_*)).

### 4.3. Synthesis of Imidazole-SH

Poly (*N*-butylimidazolium bromide) was synthesized according to a previously published method [37]. It was then mixed with equal amounts of sodium mercaptopropionate in DMF to exchange with the counter ion bromide. The product was obtained after being dialyzed (1 kDa MWCO) and freeze-dried. ^1^H NMR (500 MHz, D_2_O, d, δ (ppm): 7.47 (d, N-C*H*=C*H*-N^+^), 8.25 (d, N-C*H*=N^+^), 4.16 (t, N^+^-C*H_2_*-C*H_2_*), 1.55 (m, CH_2_-CH_2_-C*H_2_*-C*H_2_*)).

### 4.4. Synthesis of MAH-Lys

Maleic anhydride (9.8 g, 0.1 mol) and L-Lysine (14.6 g, 0.1 mol) were added in AcOH (100 mL) and stirred for 12 h at room temperature and vacuum distillation to subsequently remove the solvent. Then, the reaction solution was dispersed in 200 mL of H_2_O and heated to reflux. The white powder was collected by rotary evaporation and freeze-drying. ^1^H NMR (500 MHz, D_2_O, d, δ (ppm): 6.54 (s, C*H*=C*H*), 3.95 (t, N-C*H*-C=O), 3.03 (t, CH-C*H_2_*), 1.74 (m, C*H_2_*-C*H_2_*-C*H_2_*-N)).

### 4.5. Preparation of Antibacterial Hydrogels Containing DFO

Antibacterial hydrogels containing DFO were prepared from EPL-SH, Imidazole-SH, MAH-Lys, and DFO (2 wt% in PBS buffer). EPL-SH (0.03 g/mL), MAH-Lys (0.036 g/mL), and Imidazole-SH (0.036 g/mL) were dissolved in 1 mL of PBS buffer containing 2 wt% DFO, and the reaction was irradiated with UV light for 15 min resulting in the formation of a gel.

### 4.6. Characterization of Hydrogels

The frequency sweep (*f* = 0.1–10 Hz and *γ* = 0.5%) and amplitude sweep (*f* = 0.1 Hz and *γ* = 0.1–100%) experiments were measured by a Malvern Kinexus PRO rheometer. The surface morphology and compressive stress–strain of the freeze-dried hydrogels was observed using FE-SEM and a compression test machine (operated with a load cell of 10 N and the compression velocity was 1 mm/min).

### 4.7. Antibacterial and Anti-Inflammatory Assay of Hydrogels

Fifty microliters of a bacterial suspension (10^6^ CFU/mL of *E. coli* and *S. aureus*) were added onto the surfaces of hydrogels (El, ELI, and ELID), which were then incubated at 37 °C for 2 h. Subsequently, the hydrogels were immersed in 5 mL of PBS for 1 h to remove unattached bacteria, while attached bacteria were scraped off the surface of the hydrogels by ultrasonication for 30 min. The CFU was measured by the serial dilution method and the log reduction value, and the killing ratios were calculated by Equations (1) and (2), respectively.

(1) $${\mathrm{Log}\;\mathrm{reduction}\;\mathrm{value}=\log}_{10}[\;\frac{{\mathrm{CFU}}_{\mathrm{control}}}{{\mathrm{CFU}}_{\mathrm{sample}}}]$$

where CFU_control_ was the CFU of PBS and CFU_sample_ was the CFU of EL, ELI, and ELID.

(2) $$\mathrm{Killing}\;\mathrm{Ratio}\%=1\;-\;10^{-\mathrm{Log}\;\mathrm{reduction}\;\mathrm{value}}\;\times \;100\%$$

The antibiofilm activities of the hydrogels were evaluated by fluorescence imaging (the red fluorescence intensity was analyzed by ImageJ v1.8.0 software) and FE-SEM according to a previous report [39]. The antimicrobial activities of the hydrogels were determined by assessing their ability to inhibit bacterial growth on an agar surface. Tryptone Soy Agar (TSA) plates were seeded with *S. aureus* (10^6^ CFU/mL) and *E. coli* (10^5^ CFU/mL). The inoculated TSA plates were then covered with hydrogels and cultured at 37 °C overnight. Hydrogels lacking QA functionality were used as controls. The hydrogels were removed after 24 h. The colonies that formed under the hydrogels were counted, and the inhibition of growth zones were imaged using a digital camera.

The anti-inflammation activities of hydrogels were performed by LPS inflammation model using quantitative real-time PCR (qRT-PCR), and a detailed procedure followed a previously published report [50].

### 4.8. Hemolytic and Cytocompatibility Assay of Hydrogels

Erythrocytes were separated from the blood by centrifugation at 1200 rpm for 10 min; after that, they were washed three times with PBS buffer and then diluted to a final concentration of 5% *v*/*v*. The hydrogels were placed in a 24-well plate, of which each well was filled with 1 mL of erythrocyte solution. The plate was then shaken at 200 rpm at 37 °C for 1 h. Subsequently, the plate was centrifuged at 1500 rpm for 10 min, and the supernatant was subjected to a hemolytic activity measurement as an OD at 540 nm using a microplate reader (Infinite F50) and calculated by Equation (3).

(3) $$\mathrm{Hemolysis}\%=\frac{{\mathrm{OD}}_{\mathrm{sample}}{\;-\;\mathrm{OD}}_{\mathrm{negative}}}{{\mathrm{OD}}_{\mathrm{positive}}{\;-\;\mathrm{OD}}_{\mathrm{negative}}}\;\times \;100\%$$

The hydrogels were immersed in 5 mL of PBS for 2 h at room temperature. After that, 100 μL of the hydrogel extract was mixed with 100 μL of 10^5^ cells/well human skin fibroblast (HSF) cell suspension in a 96-well plate and incubated at 37 °C in a 5% CO_2_ atmosphere for 24 h. After 5 μL of CCK-8 reagent was added to each well, the cells were further incubated for an additional 1 h and measured OD at 540 nm using a microplate reader (Infinite F50) and calculated by Equation (4).

(4) $$\mathrm{Cell}\;\mathrm{viability}\%=\frac{{\;\mathrm{OD}}_{\mathrm{sample}\;}}{{\mathrm{OD}}_{\mathrm{control}\;}\;}\times 100\%$$

### 4.9. Cells Angiogenesis and Trasnswell Assay of HUVECs

For the angiogenesis, 100 µL of Matrigel (Becton Dickinson, Franklin Lakes, MA, USA) was added to a 48-well plate, followed by gel at 37 °C for 30 min. The 250 μL of HUVECs at a density of 3 × 10^4^ cells/cm^2^ was added onto Matrigel, and a different hydrogel degraded solution (250 μL) was used to treat the cells. Optical microscopy was employed to observe the tube formation following 8 h of incubation. The parameters and symbols of angiogenesis included nodes and tubes, which indicated the early and advanced stages, respectively.

The transwell assay was employed to assess the function of DFO in the migration of HUVECs. Briefly, 5 × 10^4^ cells/cm^2^ were seeded in the upper chamber of a 24-well transwell plate and cultured with ECM. Then, into the lower chamber, 600 µL of culture medium with different hydrogel-degraded solutions was loaded. The cells were removed from the upper chamber with a cotton swab 8 h later. Next, 4% paraformaldehyde was used to fix the cells migrating to the lower chamber, followed by 0.5% crystal violet staining for 10 min.

ImageJ v1.8.0 software was used to analyze the tube length and the number of cells migrating.

### 4.10. Statistical Analysis

The data were displayed as the mean ± standard deviation (SD), and Tukey’s post-hoc analysis measured the statistical significance. * *p* ≤ 0.05, ** *p* ≤ 0.01, *** *p* ≤ 0.001 were considered statistically significant.

## Data Availability

Not applicable.

## Supplementary Materials

The following supporting information can be downloaded at: https://www.mdpi.com/2310-2861/8/8/476/s1, Figure S1: ^1^H NMR (500 MHz, D_2_O) of EPL-SH; Figure S2: ^1^H NMR (500 MHz, D_2_O) of Imidazole-SH; Figure S3: ^1^H NMR (500 MHz, D_2_O) of MAH-Lys; Figure S4: (a) Stress–strain curve of hydrogels; (b) hemolysis of hydrogels; (c) DFO release curve.
