# Correction: Sheng et al. Antibacterial and Angiogenic Poly(ionic liquid) Hydrogels. *Gels* 2022, *8*, 476

**DOI:** 10.3390/gels9060472

**Published:** 2023-06-08

**Authors:** Chengju Sheng, Xuemei Tan, Qing Huang, Kewen Li, Chao Zhou, Mingming Guo

**Affiliations:** 1 School of Chemistry and Chemical Engineering, Southwest University, Chongqing 400715, China; scj1995@email.swu.edu.cn (C.S.); xm066248@163.com (X.T.); qinghuang3602@163.com (Q.H.); 2 College of Materials and Environmental Engineering, Hunan University of Humanities, Science and Technology, Loudi 417000, China; likewen4310@163.com; 3 Institute of Biomedical Engineering and Health Sciences, Changzhou University, Changzhou 213164, China

## Error in Figures

In the original publication [1], there were mistakes in Figures 6 and 7. We do apologize that Figures 6 and 7 were mistakes of duplicate images. We have corrected Figures 6 and 7. The corrected Figure 6 and Figure 7 appear below. The authors state that the scientific conclusions are unaffected. This correction was approved by the Academic Editor. The original publication has also been updated.

**Figure 6:**
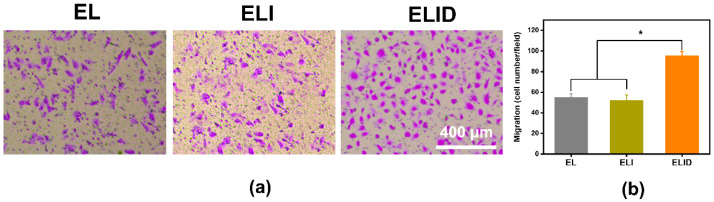
(**a**) HUVECs transwell assay of EL, ELI, and ELID (the scale bar = 400 μm); (**b**) HUVECs migration number of fields for EL, ELI, and ELID (PBS as blank and *n* = 4, * indicates *p* ≤ 0.05).

**Figure 7:**
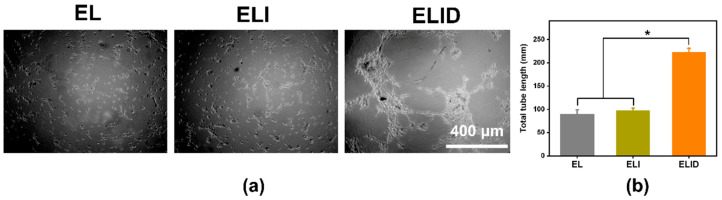
(**a**) The tube formation assay of EL, ELI, and ELID (the scale bar = 400 μm); (**b**) the total tube length of EL, ELI, and ELID (PBS as blank and *n* = 4, * means *p* ≤ 0.05).
