# Supplementary material for: Antibacterial and Angiogenic Poly(ionic liquid) Hydrogels
Source: Gels. 2022 Jul 28;8(8):476. doi: 10.3390/gels8080476 (PMC9407512; doi:10.3390/gels8080476)
Supplement: Supplementary file 1 [file gels-08-00476-s001.zip › gels-1802261-supplementary.pdf]

gels MDPI

Supplementary

Antibacterial and Angiogenic Poly(Ionic Liquid) Hydrogels

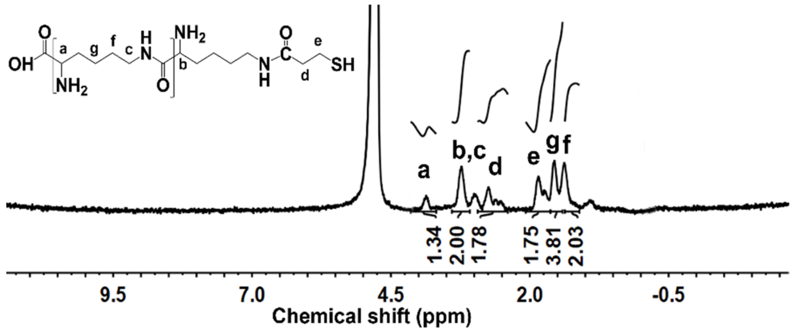

Figure S1. ^{1}H NMR (500 MHz, D_2O) of EPL-SH.

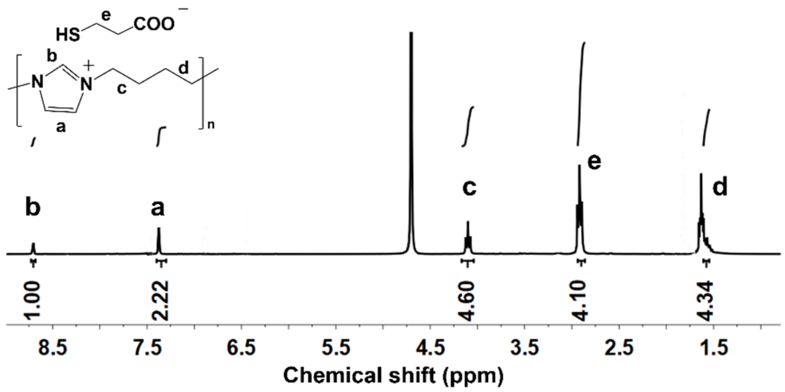

Figure S2. ^{1}H NMR (500 MHz, D_2O) of Imidazole-SH.

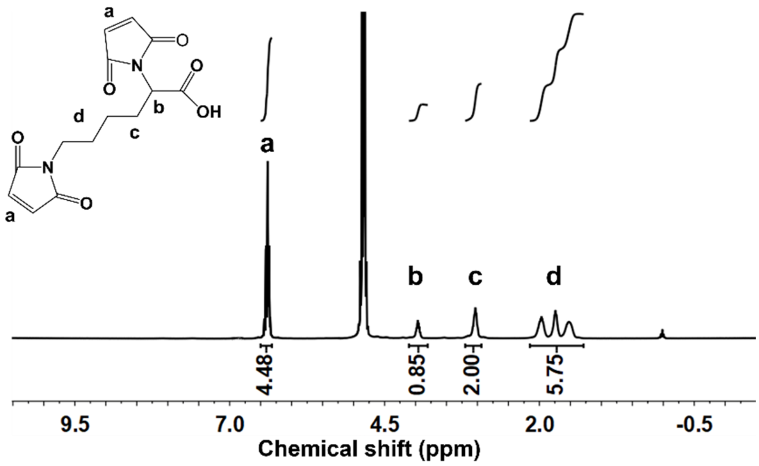

Figure S3. ^{1}H NMR (500 MHz, D_2O) of MAH-Lys.

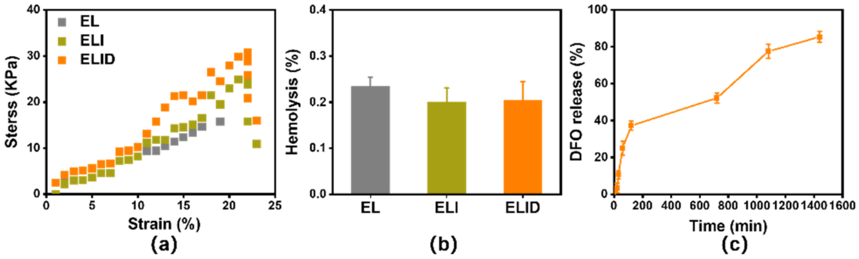

Figure S4. (**a**) Stress–strain curve of hydrogels; (**b**) hemolysis of hydrogels; (**c**) DFO release curve.
